# Supplementary material for: Single-molecule detection with a millimetre-sized transistor
Source: Nat Commun. 2018 Aug 13;9:3223. doi: 10.1038/s41467-018-05235-z (PMC6089965; doi:10.1038/s41467-018-05235-z)
Supplement: Supplementary file 1 — Supplementary Information [file 41467_2018_5235_MOESM1_ESM.docx]

**Single-molecule detection with a millimetre-sized transistor**

*Eleonora Macchia,^1^Kyriaki Manoli,^1^ Brigitte Holzer,^1^Cinzia Di Franco,^2^ Matteo Ghittorelli,^3^*

*Fabrizio Torricelli,^3^Domenico Alberga,^4^Giuseppe Felice Mangiatordi,^[[1]](#footnote-1),§^*

*Gerardo Palazzo,^1,6^Gaetano Scamarcio,^2,5^ and Luisa Torsi^1,6,7*^*

*^1^*Dipartimento di Chimica – Università degli Studi di Bari “Aldo Moro” - Bari (I)
*^2^*CNR - Istituto di Fotonica e Nanotecnologie, Sede di Bari (I)

*^3^*Dipartimento Ingegneria dell’Informazione – Università degli Studi di Brescia - Brescia (I)

*^4^*Dipartimento di Farmacia - Scienze del Farmaco – Università degli Studi di Bari “Aldo Moro” - Bari (I)

*^5^*Dipartimento di Fisica “M. Merlin” – Università degli Studi di Bari – “Aldo Moro” - Bari (I)

*^6^*CSGI (Center for Colloid and Surface Science) – Bari (I)

^7^The Faculty of Science and Engineering – Åbo Akademi University – Turku (FI)

***Supplementary Information***

***Content:***

***SN 1:*** *Surface Plasmon Resonance measurement of the SAM surface coverage p.* ***2***

***SN 2:*** *Evaluation of the SAM thickness p.* ***3***

***SN 3****: SAM surface characterization p.* ***5***

*Atomic force microscope characterization p.* ***6***

*Electrochemical characterization p.* ***8***

***SN 4:*** *The* SiMoT *current-voltage output and transfer curves p.* ***10***

***SN 5:*** *Ligand standard solutions dilution and Poisson errors p.* ***11***

***SN 6:*** *The* SiMoT *model of the FET transfer curves p.* ***12***

*Modelling of the sensing I-V transfer characteristics p.* ***12***

*Voltage trop across the SAM p.* ***16***

***SN 7:*** *The* SiMoT *model of the sensing dose-curves p.****17***

***SN 8:*** *Effect of the ionic-strength of the gating-solution on the* SiMoT *response p.* ***21***

***SN 9:*** *Quantification of endogenous human IgG in whole saliva sample p.* ***22***

***SN 10:*** *Negative control experiment in BSA p.* ***23***

***SN 11:*** *Molecular modelling of the chem-SAM p.* ***24***

*Density functional theory calculations p.* ***24***

*Molecular dynamics simulations p.* ***25***

***Supplementary References*** *p.****29*Supplementary Note 1. Surface Plasmon Resonance measurement of the SAM surface coverage**

A Surface Plasmon Resonance (SPR) Navi 200-L instrument equipped with two laser sources (670 and 785 nm wavelengths) was used to study the gold surface bio-functionalization *in situ*. Au-coated (~ 50 nm) SPR slides (BioNavis*Ltd*) comprising a chromium adhesion layer (~2 nm) provided by the vendor served as SPR substrate. The duly cleaned gold surface was functionalized with the mixed chem-SAM (3-MPA and 11-MUA, 10:1) using the protocol adopted for the gate functionalization of the single-molecule transistor (SiMoT) field-effect transistor (see Methods). After the EDC/sulfo-NHS chemical activation of the chem-SAM was accomplished, the conjugation of the bio-SAM, involving anti-Human-Immunoglobulin G (anti-IgG) and bovine serum albumin (BSA), was carried out by static injection at 22 °C of 300 μL of the relevant solutions while measuring *in situ* the optical signal changes generated by the bio-SAM formation.

The amount of capturing proteins immobilized *viz.* the surface coverage, Γ, of the SPR slide per unit area, was estimated using the Feijter formula:

$\Gamma=\frac{\left( n_{a}-n_{m} \right)d_{a}}{\frac{dn}{dc}}$ (1)

where *n_a_* is the average refractive index of the antibody layer, *n_m_* is the refractive index of the buffer solution, *d_a_*is the average layer thickness and *dn/dc* is the specific refractivity of the adsorbed antibody layer. Deriving this further to include the instrument response it returns:

$\left( n_{a}-n_{m} \right)=\Delta\theta*k$ (2)

where k is the wavelength dependent sensitivity coefficient, and Δθ is the measured angular shift. Accordingly, Supplementary Eq.1 becomes:

$\Gamma=\frac{\Delta\theta*k*d_{a}}{\frac{dn}{dc}}$ (3)

For thin layers (<100 nm) and at λ = 670 nm, the following approximations hold: k_*_d_a_ ≈ 1.0 × 10^−7^ cm/deg and *dn/dc* ≈ 0.182 cm^3^/g. Hence Supplementary Eq.3 becomes:

$\Gamma=\Delta\theta*550 ng/{cm}^{2}$ (4).

Supplementary Fig.1a shows a typical SPR sensogram obtained during the bio-conjugation process of the anti-IgG layer. When 100 μl of a 100 μg/ml anti-IgG solution in PBS is injected, an angular shift is recorded as it can be seen also in the inset of Supplementary Fig.1b. By means of Supplementary Eq.4, the surface coverage, Γ, was evaluated from the measured angular shift. The resulting Γ values are reported in Supplementary Fig. 1.1b and at the equilibrium the measured anti-IgG coverage is 283 ± 17 ng/cm^2^. Taking into account the gate electrode area (0.6 cm^2^) and the molecular weight of a single anti-IgG protein, the estimated average total number of active binding-sites is (5.92 ± 0.3) · 10^11^ so ~ 10^12^ capturing anti-IgG are packed on the gate surface. This figure is in good agreement with the measured density of conjugated antibodies Fab fragments^[[2]](#endnote-1)^ and DNA probes^[[3]](#endnote-2)^ as, for the same gate area, 7.38·10^11^ and 8.16 10^11^ bio-elements are found, respectively. The sensogram of Supplementary Fig..1 a shows also that the injection of a BSA solution (100 μl of a 100 μg/ml solution), induces very little changes in the SPR detected coverage of the SAM and hence its thickness. This feature, in agreement with the literature,^[[4]](#endnote-3)^ is explained by assuming that the BSA proteins physical adsorption fills the voids left by the previous anti-IgG bio-conjugation, making the SAM layer more compact but not thicker and yet also not affecting its ion-permeable nature (*vide infra*).


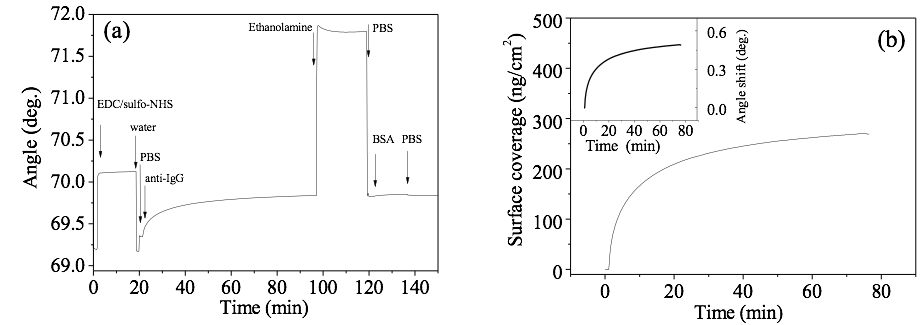


**Supplementary Figure 1:** a) Sensogram of the immobilization of anti-IgG on the Au surface pre-modified with the mixed alkanethiols chem-SAM, b) Protein surface loading vs. time. Inset: Corresponding angular response of the binding of the anti-IgG proteins as a function of time.

**Supplementary Note 2. Evaluation of the SAM thickness**

To estimate the overall SAM thickness, the SPR responses shown in Supplementary Fig..2, were fitted to a multilayer model based on the Fresnel equation using Winspall 3.02 software.^[[5]](#endnote-4)^ To start, the thicknesses and apparent refractive indexes for the Au coated SPR slide comprising the chem-SAM were measured and used to simulate the thickness changes upon anti-IgG bio-conjugation and BSA physisorption. The data coming out from the simulation of the SPR curves are presented in Supplementary Tab.2. The average 0.41 nm thickness for the chem-SAM is in line with this layer being mostly formed of short 3-mercaptopropionic acid (3-MPA) molecules. The angular shift measured upon the anti-IgG and BSA attachment, corresponds to a thickness for the bio-SAM of 5.2 nm, so as the total SAM thickness (chem-SAM + bio-SAM) on the gate is *ca.* 5.6 nm. Considering that the anti-IgG “Y-shape” has a height of 14.5 nm, a width of 8.5 nm, and a thickness of 4.0 nm,^[[6]](#endnote-5)^ the measured bio-SAM thickness is consistent with a single monolayer of capturing antibodies mostly laying edge-on the surface. Such an arrangement is demonstrated not to inhibit the capturing molecule (anti-IgG in this case) binding activity.^[[7]](#endnote-6)^

**Supplementary Figure 2:** SPR angular reflectivity curves measured at a wavelength of 670 nm for the chem-SAM (black-circles) and the SAM comprising both the chem- and bio-part (red-circles); lines are the fitting curves.

**Supplementary Table 1:** *Optical parameters used for SPR data simulation at 670 nm*

Au / chem-SAM

Au / SAM

| **Layer Number** | **Thickness [nm]** | **Refractive index (n)** | **Extinction coefficient (k)** |
| --- | --- | --- | --- |
| 1- Glass BK7 | 0 | 1.5189 | 0 |
| 2- Chromium | 1.78 | 2.5153 | 4.5721 |
| 3- Gold | 40.47 | 0.1799 | 3.8278 |
| 4- Chem-SAM | 0.41 | 1.4630 | 0 |
| 5- Bio-SAM (anti-IgG+BSA) | 5.17 | 1.4200 | 0 |
| 6- PBS | 0 | 1.3320 | 0 |

The n_SAM_ and n_anti-IgG_ values were taken from literature.^[[8]](#endnote-7),^^[[9]](#endnote-8)^

**Supplementary Note 3. SAM Surface characterization**

***Atomic force microscope characterization***

The gate topography at the different functionalization steps was measured by means of an atomic force microscope (AFM mod. NTEGRA Spectra, NT-MDT, Moscow, Russia) operated in semi-contact mode. The micrographswere recorded in air using a scan step size of 4 nm. High resolution single crystal Si probes (mod. NGS01, NT-MDT) with a resonant frequency of 150 kHz, a force constant of 5 N/m and a nominal tip radius of 10 nm, were used. The image analysis was performed by means of the Nova Px software (NT-MDT). Supplementary Fig. 3 a shows the surface topography of a representative area of a SAM surface. In Supplementary Fig. 3b the root mean squared (RMS) of surface roughness of Au, chem-SAM and SAM layers deposited on Kapton® are compared. The RMS roughness of the Au layer is related with the typical size (~ 50 nm) of the Au clusters obtained by electron beam deposition. The deposition of chem-SAM and SAM causes the increase of the RMS surface roughness by 22 % and 44 %, respectively, in accordance with reported data^[[10]](#endnote-9)^ Such morphological feature is compatible with that of a closely packed layer of Y-shaped proteins, with a preferential edge-on orientation, as the peak to valley profiles sketched in Fig.1c it reproduced.^[[11]](#endnote-10),^ ^[[12]](#endnote-11)^ This is also agreement with the whole SAM thickness measured in the previous session.


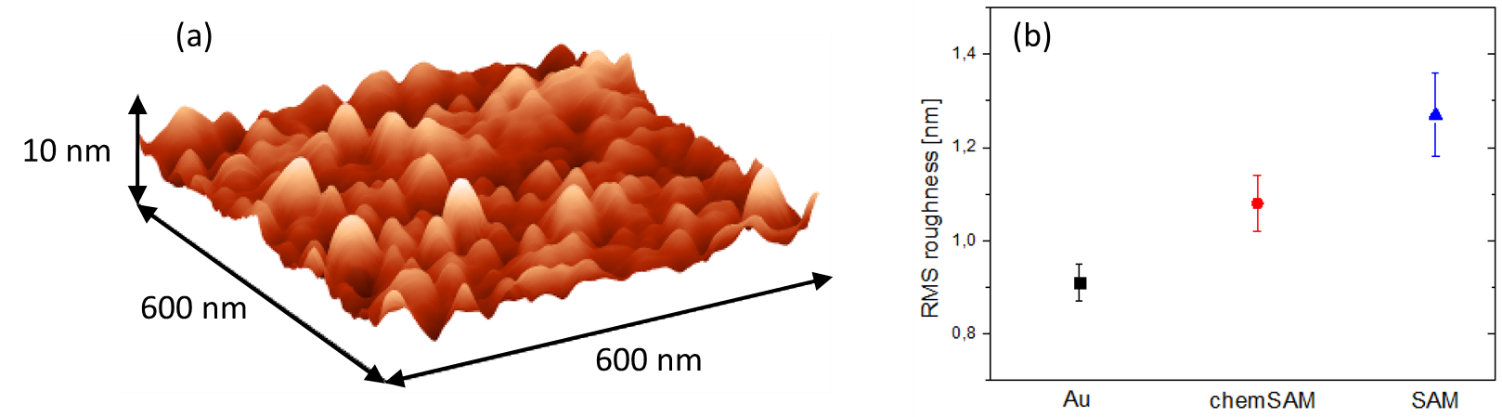


**Supplementary Figure 3:** *A representative atomic force micrograph of the SAM is shown in panel (a) while in panel (b) the root mean square (RMS) of the surface roughness is shown at different functionalization steps of the gate. The values are computed as the average over 5 representative areas while the error bars are taken as one standard deviation*

***Electrochemical characterization***

***a) Cyclic voltammetry***

A gold surfacemodified with the SAM was used as working electrode in a standard three electrode electrochemical cell. Specifically, cyclic voltammetry was carried out in the presence of 1mMK_4_[Fe(CN)_6_] · 3H_2_O (Sigma Aldrich, 98.5%) in 0.1 M potassium chloride (Fluka, puriss p.a.) in the - 0.1 to + 0.6 V potential range *vs.* an Ag/AgCl (saturated KCl) reference electrode, while the counter electrode was a gold platelet. All the electrochemical experiments were performed using a CH Instrument Electrochemical Analyser (Model CHI1230B)*.*

The changes in the faradaic response of the ferricyanide - [Fe(CN)_6_]^3-/4-^- redox probe was used to characterize the electrode ion-permeability as a function of the bio-functionalization step. Supplementary Fig.4 shows the cyclic voltammograms on the bare gold as well as after each modification step of the gold surface, namely after: - grafting of the mixed alkanethiols terminating with carboxylic functionalitiesas the EDC/sulfo-NHS chemical activation was accomplished (chem-SAM); - grafting of the anti-IgGs and finally, - after the surface blocking with ethanolamine (chemical blocking) and BSA (biological blocking). The whole process results into what is addressed as the SAM layer. The voltammograms clearly show that, after the chem-SAM immobilisation, the peak shape changed considerably. Indeed, the peak currents of the [Fe(CN)_6_]^3-^/^4-^ electrochemical reaction lowers and the peak separation increased from 73 to 170 mV (Supplementary Tab.2). The surface is therefore homogeneously covered by a layer with lower ionic permeability and hence the electron transfer is more difficult. However, while the covalent binding of the anti-IgG does not change substantially the ionic-permeability compared to the chem-SAM, the final functionalization step with ethanolamine and BSA, leads to a further current lowering and a peak separation as high as ΔV_p_ = 288 mV. This occurrence implies the presence of the anti-IgG layer on the electrode surface but, as the electrochemical activity of the [Fe(CN)_6_]^-3/-4^ redox couple is never fully suppressed, the SAM is indeed ion-permeable. Importantly, the SAM electrochemical responseto the [Fe(CN)_6_]^-3/-4^ redox couple was never affected by the interaction of the SAM with the IgG ligands at any concentration. So the binding did not appreciable changed the overall [Fe(CN)_6_]^-3/-4^ redox process nor the redox couple permeation through the SAM.


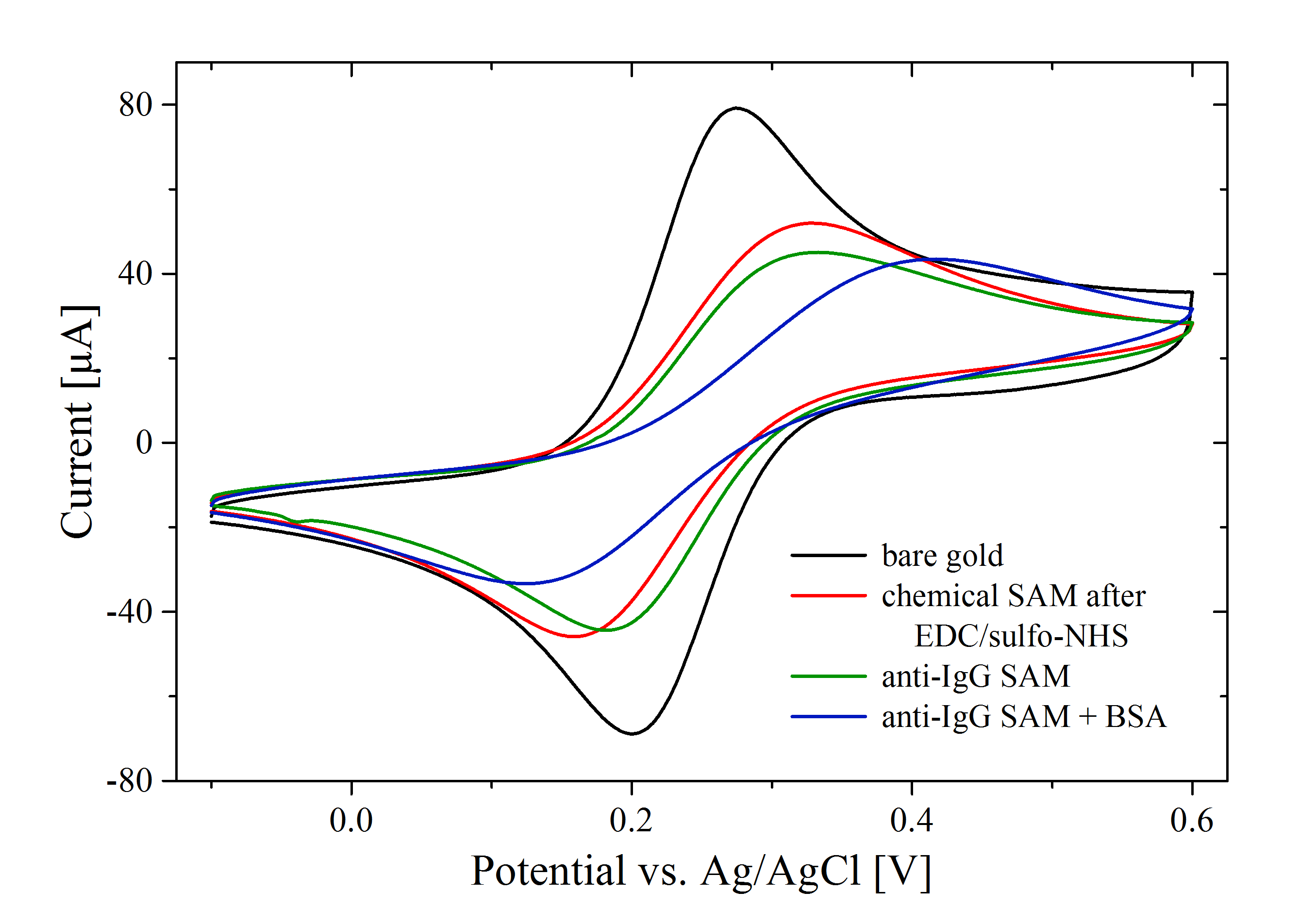


**Supplementary Figure 4:** *Cyclic voltammograms of the* [Fe(CN)_6_]^-3/-4^ redox couple *carried out at the bare gold working electrode (black-curve), after the chemical SAM grafting and activation with the EDC/sulfo-NHS (red-curve) and the anti-IgG grafting, before (green line) and after the blocking treatment in ethanolamine and BSA (blue line). Scan rate* 0.1 V s^-1^.

**Supplementary Table 2:** *Electrochemical data (cathodic peak potential, V_pc_, anodic peak potential V_pc_, and peak separation ΔV_p_,) measured in Fig.3.2S, for the bare Au as well as the chemical SAM (activated with the EDC/sulfo-NHS) and anti-IgG grafting, before and after*

*the blocking treatment in ethanolamine and BSA*

|  | V_pc_ (mV) | V_pa_(mV) | ΔV_p_(mV) |
| --- | --- | --- | --- |
| Bare gold | 272 | 199 | 73 |
| Chemical SAM | 328 | 158 | 170 |
| Anti-IgG | 330 | 184 | 146 |
| anti-IgG SAM + BSA | 413 | 125 | 288 |

It is important to add that a very weak faradaic current and a rather large capacitive current was measured when the direct electrochemical activity of the SAM electrode was inspected. This is in line with the SAM being an ion-permeablelayer composed of redox inactive species. Namely the SAM is a layer formed by fixed ions associated with the immobilized antibodies. Antibodies are indeed redox inactive species unless too high potential are applied that induces degradation processes particularly an oxidative one.^[[13]](#endnote-12)^ On the other hand, too large reductive potential can lead to the massive desorption of the chem-SAM’s chains. These detrimental effects, that can induce large instabilities in the EGOFET operation, were controlled by recording the gate current (I_G_) along with the I_D_ one.^[[14]](#endnote-13)^ When the I_G_ current increases becoming comparable to the drain current (I_D_) one, this isindicative of a large faradaic current flowing perpendicularly to I_D_, that is minimized by restricting the inspected gate voltage range to an electrochemical-reaction-free window.

***b) Electrochemical Impedance Spectroscopy analysis***

The Electrochemical Impedance Spectroscopy (EIS) analysis has been performed to measure the capacitance of the gate electrode functionalized with (i) the chemical SAM, (ii) the chemical and the biological SAM, and (iii) the capacitance of the P3HT transistor. To this end, Ag/AgCl pellets served both as counter and as reference electrodes while the working electrode is either the functionalized gate electrode or the transistor. The transistor is connected in the two-terminal configuration, *viz.* the source and drain electrodes connected together.

EIS are performed as a function of the DC voltage. At each operating voltage a sinusoidal signal with an amplitudeof 20 mV is applied. A frequency range from 1 Hz to 10k Hz is investigated while the electrolyte solution is NaCl 0.01 M. The capacitance is obtained by modeling the impedance spectra with the Randless equivalent circuit.^[[15]](#endnote-14)^ In all the investigated cases the charge transfer resistance and the Warburg element could be neglected, while the constant phase element yields the electric-double-layer (EDL) capacitance. The extracted capacitance as a function of the applied DC voltage is shown in Supplementary Fig. 5 for the gate functionalized with the chem-SAM. The capacitance slightly decreases by decreasing the applied voltage and a plateau is displayed at V_DC_ = -0.2 V. The mean value of the total capacitance is 6μF. Since from Supplementary Table 2 the chem-SAM thickness is equal to 0.41 nm, we can estimate a relative permittivity of the chem-SAM to be equal to 4.6. This further confirms that the chem-SAM is rather impermeable to the electrolyte ions.

**Supplementary Figure 5:** *Capacitance of the gate electrode functionalized with the chem-SAM as a function of the applied DC voltage*.

We performed the EIS analysis by considering the gate functionalized with the SAM and, in this case, we found a gate capacitance equal to 6.5 μF. The slight increase of the capacitance (less that 10%) could be attribute to the process variability. The results confirm that the bio-SAM, being ion permeable, holds a capacitance higher that of the chem-SAM ones. This results in a total SAM capacitance equal to C’_G_ = 6 μF, that normalized to the gate area (*viz.* 0.6 cm^2^) yields a capacitance per unit area C_G_ = 10 μF/cm^2^.

To compare the capacitance of the gate electrode with that of the transistor, we performed EIS on the P3HT transistor using the very same experimental set-up and geometries adopted during the EGOFET operation. Supplementary Fig. 6 shows the transistor capacitance as a function of the applied DC voltage. As expected the capacitance increases by increasing the applied gate voltage (absolute value) because a larger charge carrier concentration is accumulated at the electrolyte/semiconductor interface. The maximum capacitance is equal to C_OSC_ = 7 0nF at V_DC_ = -0.4 V, which is about two orders of magnitude lower than the whole capacitance of the functionalized gate electrode. In the case of the P3HT transistor, the area is equal to 6.4 10^-3^ cm^2^, which results in a capacitance per unit area C’_OSC_ = 11μF/cm^2^.

This analysis shows that the EDL capacitance per unit area obtained at the SAM interface is comparable with the maximum EDL capacitance obtained at the transistor interface and, as a consequence, C_GATE_ = 100 × C_OSC_as A_GATE_ = 100 × A_OSC_.

**Supplementary Figure 6:** *Capacitance of the P3HT transistor as a function of the applied DC voltage.*

**Supplementary Note 4. The SiMoT current-voltage output and transfer curves**

Typical SiMoT output characteristics showing the drain current (I_D_) measured as a function of V_D_ at gate voltages, V_G,_ ranging between 0 and - 0.5V, in steps of - 0.1 V are shown in Suppl. Fig.7 a, while the I_D_ – V_G_ transfer curves at V_D_ = - 0.4 V are shown in Fig. 7 b. The curve in the inset of Suppl. Fig.7 b is the gate current I_G_, that is at least two orders of magnitude lower than I_D_. The curves were measured in the forward and reverse mode to evidence the occurrence of hysteresis, that was minimized by tuning the inspected voltage ranges away from the potential where electrochemical process occurred.

**Supplementary Figure 7:** *(a) Typical output characteristics I_D_-V_D_ of the* SiMoT*. (b) Typical transfer characteristics I_D_-V_G_ of the* SiMoT *comprising a bare Au gate (black and blue curves) or the SAM one (red-curve). The black and blue curves are taken on the same* SiMoT *before and after measuring the whole sensing calibration curve shown in Fig. 2d. The I_G_ for the SAM gate is shown in the inset.*


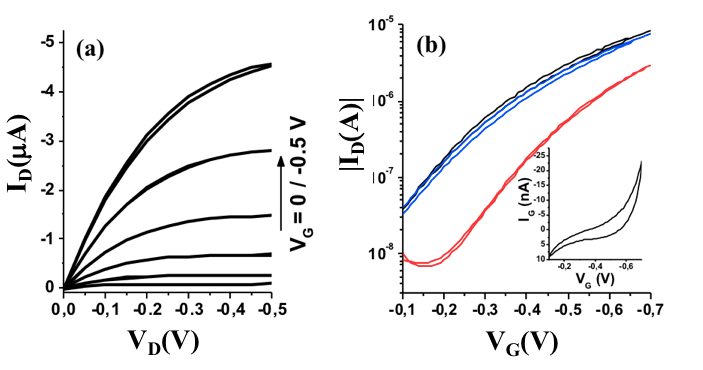


The black curve in Suppl. Fig.7b is the current measured on a bare gold gate after the stabilizationof the organic semiconductor, carried out by cycling the transistor in the range - 0.1 < V_G_< - 0.7 V in water, until the current overlays previous traces for at least three times. The current measured when the gate is functionalized with the SAM, is shown in red. A shift of the threshold voltage (V_T_) or, equivalently, of the gate work function,  of about 0.3 is found. The blue curve is the current measured on the same device comprising the gold gate used to measure the black curve, but after the acquiring of a whole IgG dose curve, such as the ones shown in Fig. 3 of the main text. As it is evident the blue and the black curves almost overlap. This control experiment is important as the SAM functionalized gate undergoes an irreversible change of its electrostatic properties after the assay,being the sensing process irreversible. Hence, the level of I_D_ flowing in the P3HT channel needs to be controlled by means of an independent reference gate before and after the sensing. The degree of change of the I_D_ current level was deemed acceptable only when lower than 8%.

**Supplementary Note 5. Ligand standard solutions dilution and Poisson errors**

The IgG or IgM PBS standard solutions, at a nominal concentrations ranging from 6·10^-2^zM to 6·10^8^zM (1 zM = 10^-21^ mole · L^-1^), were prepared by a serial dilution process according to the following equation: ***c_n_= c_n-1_ V_n-1_/ V_n_= k · c_n-1_***. Here **c_n-1_** and **c_n_** being the ligand concentrations in the stock and in diluted solution, respectively, while **V_n-1_**and **V_n_** are the corresponding solution volumes and ***k = V_n-1_ / V_n_*** is the dilution factor. As customary, the former dilution is the stock solution for the subsequent dilution in the series. The absolute uncertainty of the concentration for each standard solution was computed as the propagation error of the dilution factor, while the maximum uncertainty of the volume, given by the supplier company of the pipettes used, is 1%. This value of the uncertainty of the volume takes into account both random and systematic errors in pipetting. The nominal number of IgG or IgM proteins (# IgX) at each concentration is given by:

$\boldsymbol{\# IgX=cV}\boldsymbol{N}_{\boldsymbol{A}}$ (5)

where **c** is the ligand concentration (in molarity), **V** is the volume of the solution in which the gate is incubated (100 μL) and **N_A_** is the Avogadro number. The uncertainty associated with the sampling in the serial dilution can be estimated, according to the Poisson’s distribution, as the square root of the expected number of IgX proteins corresponding to one standard deviation. The total uncertainty of the ligand concentration has been evaluated as the square root of the sum of the squares (RSS) of the dilution (**σ_D_**) and Poisson’s **(σ_P_**) errors,$\sigma_{RSS}=\sqrt{\sigma_{D}^{2}+\sigma_{P}^{2}}$. The relevant error bars, used thorough the present study, are shown in Supplementary Tab.3.

**Supplementary Table 3.** *The ligand solutions uncertainty is computed as the* $\sigma_{RSS}$ *error value. The ligand nominal concentration (zM), the number of proteins (# IgX) and the Poisson error (σ_P_), taken as one standard deviation, are also shown*

| **[ligand] (zM)** | **σ_RSS_ (zM)** | **# IgX** | **σ_P_ (#)** | **[ligand] (zM)** | **σ_RSS_ (zM)** | **# IgX** | **σ_P_ (#)** |
| --- | --- | --- | --- | --- | --- | --- | --- |
| 0.06 | 0* | 0 | 0 | 600 | 100 | 39 | 6 |
| 0.6 | 0* | 0 | 0 | 6.7 · 10^3^ | 0.3 · 10^3^ | 392 | 20 |
| 6 | 0* | 0 | 0 | 6.7 · 10^4^ | 0.1 · 10^4^ | 3.92 10^3^ | 60 |
| 10 | 5 | 1 | 1 | 6.67 · 10^5^ | 0.03 · 10^5^ | 3.92 10^4^ | 2 · 10^2^ |
| 20 | 10 | 1 | 1 | 6.67 · 10^6^ | 0.02 · 10^6^ | 3.92 10^5^ | 6 · 10^2^ |
| 30 | 15 | 2 | 1 | 6.67 · 10^7^ | 0.02 · 10^7^ | 3.92 10^6^ | 2 · 10^3^ |
| 60 | 30 | 4 | 2 | 6.67 · 10^8^ | 0.01 · 10^8^ | 3.92 10^7^ | 6 · 10^3^ |
| 100 | 33 | 6 | 2 |  |  |  |  |

* the error is taken as the sole Poisson one

**Supplementary Note 6. The SiMoT model of the FET transfer curves**

***Modelling of the sensing I-V transfer characteristics***

In high-molecular-weight polymers charge transport occurs through an interconnected network of ordered regions while the amorphous fraction of the film does not participate to the transport. As charges reside in the ordered regions, the structural disorder in these regions define the electronic properties and it can be quantitatively measured by the paracristallinity parameter *g.*^[[16]](#endnote-15)^More in detail, in the case of poly(3-hexyl-thiophene), P3HT – a semicrystalline conjugated polymer – Noriega *et al.*found an intermediate paracristallinity regime (g ~ 3-7%) indicating the coexistence of localized and delocalized states: in paracrystalline aggregates the charge is transported by a mechanism where mobile charge is temporarily trapped in localized states, akin the multiple trapping and release.^[[17]](#endnote-16),^ ^[[18]](#endnote-17),^^[[19]](#endnote-18),^ ^[[20]](#endnote-19),^ ^[[21]](#endnote-20),^^[[22]](#endnote-21)^

Based on the elicited studies, here we derive a current model of SiMoTs accounting for the multiple trapping and release transport mechanism and an exponential tail trap density of states.^,,^The drain-source current flowing in the semiconductor can be calculated by solving the drift-diffusion transport equation:^[[23]](#endnote-22)^

| $\boldsymbol{I}_{\boldsymbol{D}}\boldsymbol{=}\frac{\boldsymbol{W}}{\boldsymbol{L}}\int_{\boldsymbol{V}_{\boldsymbol{S}}}^{\boldsymbol{V}_{\boldsymbol{M}}} \int_{\boldsymbol{V}_{\boldsymbol{ch}}}^{\boldsymbol{\varphi}_{\boldsymbol{s}}} \frac{\boldsymbol{\sigma}\left( \boldsymbol{\varphi,}\boldsymbol{V}_{\boldsymbol{ch}} \right)}{\boldsymbol{F}_{\boldsymbol{x}}\left( \boldsymbol{\varphi,}\boldsymbol{V}_{\boldsymbol{ch}} \right)}\boldsymbol{d\varphi d}\boldsymbol{V}_{\boldsymbol{ch}}$ | (6) |
| --- | --- |

where W is the transistor width, L is the transistor length, V_S_ is the source voltage, V_M_ = min{V_D_, V_G_-V_T_} V_D_ is the drain voltage, V_G_ is the gate voltage, and V_T_ is the threshold voltage, that in our model is equal to the flat-band voltage. V_ch_ is the potential along the channel (*i.e.* the Pseudo-Fermi potential), φ is the surface potential, and σ is the hole conductivity:^,,,,,^

| $\boldsymbol{\sigma=q}\boldsymbol{\mu}_{\boldsymbol{0}}\boldsymbol{p}_{\boldsymbol{0}}\boldsymbol{exp}\left[ \frac{\boldsymbol{q}\left( \boldsymbol{\varphi-}\boldsymbol{V}_{\boldsymbol{ch}} \right)}{\boldsymbol{k}_{\boldsymbol{B}}\boldsymbol{T}} \right]$ | (7) |
| --- | --- |

q is the elementary charge, μ_0_ is the hole mobility in the delocalized states, p_0_ = N_HOMO_ exp[E_G_/(2k_B_T)] is the intrinsic hole concentration into the semiconductor, N_HOMO_ is the total density of delocalized states in the highest occupied molecular orbital (HOMO) levels, E_G_ = E_LUMO_-E_HOMO_ is the energy gap, E_LUMO_ is the lowest unoccupied molecular orbital energy level, E_HOMO_ is the HOMO energy level, k_B_ is the Boltzmann constant, and T is the temperature. F_x_ is the electric field into the P3HT, which is orthogonal to the electrolyte-semiconductor interface. Under the gradual channel approximation (dF_x_/dx >>dF_y_/dy), which is valid for SiMoTs, F_x_ can be calculated as follows:

| $\boldsymbol{F}_{\boldsymbol{x}}\left( \boldsymbol{\varphi,}\boldsymbol{V}_{\boldsymbol{ch}} \right)\boldsymbol{=}\left[ \frac{\boldsymbol{2}\boldsymbol{q}}{\boldsymbol{\epsilon}_{\boldsymbol{s}}}\int_{\boldsymbol{V}_{\boldsymbol{ch}}}^{\boldsymbol{\varphi}} \boldsymbol{p}\left( \boldsymbol{\xi,}\boldsymbol{V}_{\boldsymbol{ch}} \right)\boldsymbol{d\xi} \right]^{\boldsymbol{1/2}}$ | (8) |
| --- | --- |

where ε_s_ = ε_0_ κ_s_ is the semiconductor permittivity, ε_0_ is the vacuum permittivity, κ_s_ is the relative permittivity of the P3HT, the variable ξthat indicates a potential and p is the hole concentration, that can be calculated as follows:

| $\boldsymbol{p(}\boldsymbol{E}_{\boldsymbol{F}}\boldsymbol{)=}\int_{\boldsymbol{-\infty}}^{\boldsymbol{+\infty}} \boldsymbol{g}\left( \boldsymbol{E} \right)\left[ \boldsymbol{1-}\boldsymbol{f}_{\boldsymbol{d}}\left( \boldsymbol{E,}\boldsymbol{E}_{\boldsymbol{F}} \right) \right]\boldsymbol{dE}$ | (9) |
| --- | --- |

E is the energy defined with respect to E_HOMO_, E_F_ = E_HOMO_ – E_LUMO_ + q(φ - V_ch_) is the Fermi energy level in the organic semiconductor, g(E) = g_t_(E) + g_b_(E) is the total density of states and accounts for both the tail trap states g_t_ = (N_t_/E_t_) exp[(E-E_HOMO_)/(E_t_)], E < E_HOMO_ and the delocalized states g_b_(E) = N_HOMO_/(k_B_T) [(E-E_HOMO_)/(k_B_T)]^(1/2)^, E > E_HOMO_, N_t_ is the total density of tail localized states and E_t_ is the energy disorder.^,,,^f_d_(E,E_F_) = {1 + exp[(E-E_F_)/(k_B_T)]}^-1^ is the Fermi-Dirac occupation probability.

Applying Gauss’ law to the water/semiconductor interface, the electric field into the semiconductor results F_x_(φ_s_) = (C_OSC_/ε_s_)(V_EL_ - φ_s_), where C_OSC_ = ε_0_κ_w_/t_OSC_ is the water/semiconductor electric-double-layer capacitance per unit area, κ_w_ is the relative permittivity of water, t_OSC_ is the thickness of the electric-double-layer at the water/semiconductor interface. The potential of the electrolyte solution can be calculated as V_EL_ = (C_G_A_G_/C_T_)(V_G_ – V_T_) + (C_OSC_A_OSC_/C_T_)φ_s_, where C_G_ = (ε_0_κ_SAM_/t_SAM_) is the gate/water capacitance per unit area, κ_SAM_ and t_SAM_ are the relative permittivity and the thickness of the chem-SAM self-assembled monolayer, respectively, and C_T_ = C_G_A_G_ + C_SAM_A_OSC_. The measured capacitances result C’_G_ = C_G_A_G_ = 6 F and C’_OSC_ = C_OSC_A_OSC_ = 69 nF so therefore V_EL_ can be approximated as V_EL_= (C_G_A_G_/C_T_) (V_G_ – V_T_). Finally, φ_s_ can be calculated form the continuity of the displacement at the electrolyte-semiconductor interface:

| ${\frac{\mathbf{C}_{\mathbf{OSC}}}{\boldsymbol{\varepsilon}_{\mathbf{s}}}\left( \mathbf{V}_{\mathbf{G}}\mathbf{-}\mathbf{V}_{\mathbf{T}}\mathbf{-}\boldsymbol{\varphi}_{\mathbf{s}} \right)\mathbf{=}\left[ \frac{\mathbf{2q}}{\boldsymbol{\epsilon}_{\mathbf{s}}}\int_{\mathbf{V}_{\mathbf{ch}}}^{\boldsymbol{\varphi}_{\mathbf{s}}} \mathbf{p}\left( \boldsymbol{\xi,}\mathbf{V}_{\mathbf{ch}} \right)\boldsymbol{d\xi} \right]}^{\mathbf{1/2}}$ | (10). |
| --- | --- |

For each set {V_G_, V_D_, V_S_} the integral expression of the drain current, valid in all the regions of operation, is calculated by inserting Suppl. Eq. 7 – Suppl. Eq. 9 in Suppl. Eq.6, and the surface potential is calculated with Suppl. Eq.10. The geometrical and physical model parameters are listed in Suppl. Tab.4 and are obtained as follows.

**Supplementary Table 4.** *Geometrical and physical parameters of the drain current model*

| **Parameter** | **Value** | **Note** |
| --- | --- | --- |
| Width | W = 1280 μm | Measured |
| Length | L = 5 μm | Measured |
| Organic semiconductor area | A_OSC_ = 6.4 10^-3^ cm^2^ | Measured |
| Gate area | A_GATE_ = 0.6 cm^2^ | Measured |
| Semiconductor permittivity | ε_s_ = 3 | Suppl. Ref . |
| Maximum hole field-effect mobility | μ_0_ = 5.8 10^-2^ cm^2^ V^-1^ s^-1^ | Fitted |
| Lowes Unoccupied Molecular Orbital energy level P3HT | E_LUMO_ = 3.2 eV | Suppl. Ref. |
| Highest Occupied Molecular Orbital energy level P3HT | E_HOMO_ = 5.1 eV | Suppl. Ref. |
| Total density of HOMO states | N_HOMO_ = 1.28 10^20^ cm^-3^ | Suppl. Refs., |
| Temperature | T = 298 K | Measured |
| Total density of trap tail states | N_t_ = 1.75 10^20^ cm^-3^ | Fitted |
| Energy width of the trap tail states | E_t_ = 72 10^-3^eV | Fitted |
| Electric double layer (EDL) capacitance | C’_OSC_ = 69 nF | Measured |
| EDL capacitance per unit area | C_OSC_ = 11μF cm^-2^ | Measured |
| Gate SAM capacitance | C’_G_ = 6 μF | Measured |
| Threshold (flat-band) voltage baseline | V_T_ = - 0.24 V | Fitted |

W, L, A_OSC_, A_G_, C’_G_, C’_OSC_and T are measured. The values of κ_s_ = 3, E_LUMO_ = 3.2 eV, and E_HOMO_ = 5.1 eV are taken from Suppl. Ref. ^[[24]](#endnote-23)^. The total density of states N_HOMO_ = 1.28 10^20^ cm^-3^ is taken from the density functional theory calculations.^,^^[[25]](#endnote-24)^ C_OSC_ in the case of P3HT/water interface is found in the range 3-6 μF cm^-2^ in Suppl. Ref. ^[[26]](#endnote-25)^ and 10.8 ± 1.2 μF cm^-2^ in Suppl. Ref. ^[[27]](#endnote-26)^, depending on the P3HT surface roughness, and we measured a value of C_OSC_ = 11μF cm^-2^. Finally, the hole mobility in the delocalized states μ_0_, the total density of tail localized states N_t_, the energy disorder width E_t_, and the threshold voltage V_T_ are obtained by fitting the drain current model (Suppl. Eq.7S and Suppl. Eq.10S) with the transfer characteristics of an SiMoT incubated with PBS (baseline). We obtained μ_0_ = 0.058 cm^2^ V^-1^ s^-1^, N_t_ = 1.75 10^20^ cm^-3^, E_t_ = 72 meV, and V_T_ = 0.237 V. It is worth noting that all the extracted parameters are in very good agreement with previous work on P3HT transistors.^,^ According to Suppl. Refs., ^[[28]](#endnote-27)^ the extracted E_t_ yields a paracristallinity g ~ 5%, which is fully consistent with the multiple trapping and release transport model here used.

Then, we adopted the model to predict the drain current of the SiMoTs as a function of the ligand concentration ***c***. By varying ***c*** we found that only the threshold voltage V_T_ changes while all the other model parameters are constant. As shown in Supplementary Fig.8 and Supplementary Fig.9, the model (dashed-black-line) accurately predicts the measurements (full-color-lines) in the whole range of gate voltage and ligand concentration. To further corroborate the model, in Supplementary Fig.10 we compare the field-effect mobility calculated from the measured transfer characteristics (full-color-line) with that predicted by the model (dashed-black-line). The comparison between the extracted and predicted mobility confirms the accuracy of the model in the whole range of gate voltages and ligand concentrations.

Relevantly, the gate leakage current does not affect the extraction of the threshold voltage. This has been assessed by modelling the I_D_-V_G_ characteristics as a function of the ligand concentration by subtracting the measured gate current (I_G_) from the total drain current (I_D_). The parameters of the model are the very same of those obtained including the gate leakage current and reported in Table 4S.

**Supplementary Figure 8: *Modeling of*** *the* ***SiMoT characteristics.*** *Measured I-V characteristics (full-line) and model (dashed-line) as a function of the ligand concentration (in semi-log scale)*

**Supplementary Figure 9: *Modeling of the SiMoT characteristics.*** *Measured I-V characteristics (full-line) and model (dashed-line) as a function of the ligand concentration (in linear scale).*

**Supplementary Figure 10: *Field-effect mobility of SiMoT.*** *Field-effect mobility calculated from the I-V measurements (full-line) and predicted by the model (dashed-line) as a function of the ligand concentration. The Field-effect mobility is calculated in saturation region (V_D_ = -0.4 V, V_T_ ≤ -0.237 V) as follows: μ_FE_ = (d√I_D_/dV_G_)^2^/(W/L C_OSC_). The model parameters are listed in Tab.6.1S.*

***Voltage drop across the SAM***

***
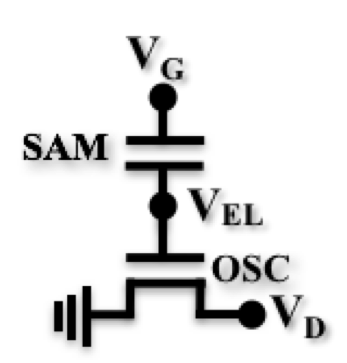
***

***Suppl. Figure 6.4S:*** *Simplified equivalent circuit for the*

*SiMoT device*

The FET is gated thought the series of the water/semiconductor electric-double-layer (EDL) capacitance (C’_OSC_) and the gate/water EDL capacitance (C’_G_). The potential in the solution (V_EL_) lays between (V_G_– V_T_) and V_D_. Considering the system as a capacity divider, the following holds:

$V_{EL}= \frac{(V_{G} -V_{T})\cdot C_{G}^{'}}{C_{G}^{'} + C_{OSC}^{'}} + \frac{V_{D}\cdot C_{OSC}^{'}}{C_{G}^{'} + C_{OSC}^{'}}$ (11)

For V_D_ = 0: $V_{EL}= \frac{(V_{G} -V_{T})\cdot C_{G}^{'}}{C_{G}^{'} + C_{OSC}^{'}}$ (12 ).

The $C_{OSC}^{'}<C_{G}^{'}$ is a consequence of the gate being much larger than the channel area as well as of the bio-SAM layer being an ionic conductor, so that the low capacitive coupling between the gate and the OSC turns into a FET that is modulated very well by the charge variations on the gate. In other words, the device is by design extremely sensitive to electrostatic changes in the SAM and hence only V_T_ changes are measured. The potential drop within the SAM layer ΔV_SAM_ is give by:

${\Delta V}_{SAM}=\left[ \left( V_{G}-V_{T} \right)- V_{EL} \right]=\left[ \left( V_{G}-V_{T} \right) - \frac{\left( V_{G}- V_{T} \right)\cdot C_{G}^{'}}{C_{G}^{'} + C_{OSC}^{'}} \right]=\left( V_{G}-V_{T} \right)\cdot\frac{C_{OSC}^{'}}{C_{G}^{'}+ C_{OSC}^{'}}$ (13)

Considering the measured capacitance, ΔV_SAM_≈ 10^-2^ (V_G_ –V_T_) hence at V_G_ = - 0.7 V and a V_T_ = - 0.2 V a potential drop of ≈ 5 mV occurs in the SAM resulting in an electric field of ≈ 10^4^ V/cm.

**Supplementary Note 7. The SiMoT model of the sensing dose curves**

The aim of this section is to find an analytical function that models the calibration curves measured with the SiMoT. The model is based on the Poisson distribution probability to better account for the occurrence of few binding events. It alsoprovides a rationale for the amplification mechanismoccurring in the SAM that enables the single-molecule detection.

Let’s consider a gate that comprises a compact SAM uniformly covering the gate area with a number ***n***=10^12^ of anti-IgG capturing antibodies. The Poisson distribution probability ***P_k_*** that a number ***k*** of IgG/anti-IgG binding events occur is:

$\boldsymbol{P}_{\boldsymbol{k}}\boldsymbol{=}\frac{\boldsymbol{\lambda}^{\boldsymbol{k}}\boldsymbol{e}^{\boldsymbol{-}\boldsymbol{\lambda}}}{\boldsymbol{k}\boldsymbol{!}}$ (14)

where $\boldsymbol{\lambda}$ is the average number of affinity ligands available per binding or capturing site. ***N*** is the number of ligands in the volume ***V*** of an incubation solution at a nominal concentration ***c (M)***, hence:

$\boldsymbol{\lambda=}\frac{\boldsymbol{N}}{\boldsymbol{n}}\boldsymbol{=}\frac{\boldsymbol{c V}\boldsymbol{N}_{\boldsymbol{A}}}{\boldsymbol{n}}$(15)

N_A_ being the Avogadro’s number. The probability that a given binding site does not interact with any ligand is derived from Supplementary Eq. 14 at ***k*** = 0:

$\boldsymbol{P}_{\boldsymbol{0}}\boldsymbol{=}\boldsymbol{e}^{\boldsymbol{-}\boldsymbol{\lambda}}$ (16).

Let’s assume that the SAM on gate is divided into a number of domains. Each domain comprises a given number of capturing anti-IgGs, characterized by the property that, if one IgG binds to any of the anti-IgGs in a given domain, the entire domain changes its work-function  due to a propagation effect. The propagation effect is mediated by the cooperative interactions enabled by the hydrogen bonding (H-bond) network only when it is immersed into the gating field. The process is irreversible so as no other change in  is possible, within that domain, if other affinity bindings occur. Reasonably, the propagation of the -changing effect needs a compact and uniform SAM (both the chemical and the biological components), because the presence of a defect will stop the propagation and limit the domain. Relevant to outline is that the compact nature and the defectsof the SAM are here considered only as associated with the electrostatic properties of the SAM. Under such assumptions, a given SAM ischaracterizedby a distribution of domains of different sizes, or equivalently, by a distribution of domains including a given number ofcapturing sites that will all change their work function when at least one IgG binds. The number of binding sites within a domain is ***x***. Clearly, the more the SAM is compact and defect-free the larger the domaingenerated upon interaction with one IgG is. Under the assumption that the probability of interaction of each of the ***k*** lgG ligands, with each of the ***x*** anti-IgG capturing sites is mutually independent, the probability $\boldsymbol{f}_{\boldsymbol{0}}\left( \boldsymbol{x,\lambda} \right)$that none of these domains interacts with anyone of the ***k*** ligands, is given by the product of the probabilities for each binding site to remain empty, so that:

$\boldsymbol{f}_{\boldsymbol{0}}\left( \boldsymbol{x,\lambda} \right)\boldsymbol{=}\prod_{\boldsymbol{1}}^{\boldsymbol{x}} \boldsymbol{P}_{\boldsymbol{0}}\boldsymbol{=}\boldsymbol{P}_{\boldsymbol{0}}^{\boldsymbol{x}}\boldsymbol{=}\boldsymbol{e}^{\boldsymbol{-\lambda x}}$ (17).

Let’s call ***Ψ(x)*** the yet unknown probability distribution function of the number of binding sites ***x*** (anti-IgG) found in each domain forming the SAM. The overall probability of finding a domain of anti-IgGs that has not interacted with any ligand upon exposure to the ***N*** ligands present in the incubation volume ***V,*** or equivalently to a solution of a given concentration ***c***, can be calculate from Supplementary Eq.17 as follows:

$\boldsymbol{f}_{\boldsymbol{0}}\left( \boldsymbol{\lambda} \right)\boldsymbol{=}\int_{\boldsymbol{1}}^{\boldsymbol{n}} \boldsymbol{\Psi}\boldsymbol{(x)}\boldsymbol{f}_{\boldsymbol{0}}\left( \boldsymbol{x,\lambda} \right)\boldsymbol{dx}\boldsymbol{=}\int_{\boldsymbol{1}}^{\boldsymbol{n}} \boldsymbol{\Psi}\boldsymbol{(x)}\boldsymbol{e}^{\boldsymbol{-\lambda x}}\boldsymbol{dx}\boldsymbol{\approx}\int_{\boldsymbol{0}}^{\boldsymbol{\infty}} \boldsymbol{\Psi}\boldsymbol{(x)}\boldsymbol{e}^{\boldsymbol{-\lambda x}}\boldsymbol{dx}$ (18).

The upper limit of integration, ***n*** = 10^12^, has been reasonably approximated to infinite and the lower one to zero, so thatthe probability $\boldsymbol{f}_{\boldsymbol{0}}\left( \boldsymbol{\lambda} \right)$ in Eq.18 is the Laplace Transform of the distribution function ***Ψ(x)*** of the number of binding sites ***x*** in each domain of a given SAM. Hence, ***Ψ(x)*** describes the domains sizes distribution. Indeed, the probability that one ligand binds to at least one domain is:

$\boldsymbol{f}_{\boldsymbol{k\geq1}}\left( \boldsymbol{\lambda} \right)\boldsymbol{=1-}\boldsymbol{f}_{\boldsymbol{0}}\left( \boldsymbol{\lambda} \right)$ (19).

Each data point in a given ***ΔI/I*** dose curve, as for instance those reported in Suppl. Fig. 14 or Fig. 3a in the main text, is proportional to the number of domains in which the work-function has been switched by the binding with at least one IgG ligand (at a given concentration ***c***); so the following holds:

$\frac{\boldsymbol{\Delta I}}{\boldsymbol{I}_{\boldsymbol{0}}}\boldsymbol{=}\boldsymbol{A}_{\boldsymbol{sat}}\left( \boldsymbol{1-}\boldsymbol{f}_{\boldsymbol{0}}\left( \boldsymbol{\lambda} \right) \right)$ (20).

with ***A_sat_*** being the ***ΔI/I*** saturation value. The dependence of ***ΔI/I*** from the IgG concentration ***c*** through ***λ*** (Suppl. Eq.15). ***Ψ(x)*** could be obtained from the inverse Laplace Transform of the experimental data ***ΔI/I*** set in the ***λ*** or ***c*** domain, enabling the best reproduction of the dose-curve data. Unfortunately, the inversion of the Laplace Transform is ill-conditioned and, although several alternative numerical methods have been proposed,^[[29]](#endnote-28)^ the use of a model function to fit the experimental data in  is here preferred. Hence, a suitable probability distribution needs to be found to analytically reproduce the unknown ***Ψ(x)***.

The gamma-distribution is a two-parameter~~-~~probability distribution function. The exponential and the χ-squared distributions are special cases of the gamma-distribution. Such a distribution, maximizing entropy, minimizes the amount of *a-prior* information built into the distribution. Moreover, it is widely applicable as many systems tend to move towards maximal entropy configurations over time. Indeed, such a distribution has been widely used to describe also the kinetics in systems that present a distribution of rate constants,^[[30]](#endnote-29),^^[[31]](#endnote-30),^^[[32]](#endnote-31),^^[[33]](#endnote-32)^as in the case under study. The unknown ***Ψ(x)*** distribution is therefore approximated by a ***unimodal gamma-distribution***^[[34]](#endnote-33)^*i.e.*

$\boldsymbol{\Psi}\left( \boldsymbol{x} \right)\boldsymbol{=}\frac{\boldsymbol{x}^{\boldsymbol{b-1}}}{\boldsymbol{K}^{\boldsymbol{b}}}\frac{\boldsymbol{exp}\left( \boldsymbol{-}\frac{\boldsymbol{x}}{\boldsymbol{K}} \right)}{\boldsymbol{\Gamma}\boldsymbol{(b)}}$ (21)

Where ***Γ(b)*** is the ***gamma function.*** The ***Ψ(x)*** characteristic shape (**b**) and scale (***K)*** parameterscorrelate to the distribution mean as $\bar{\boldsymbol{x}}\boldsymbol{=Kb}$ and the variance $\boldsymbol{\sigma}^{\boldsymbol{2}}\boldsymbol{=b}\boldsymbol{K}^{\boldsymbol{2}}$. As for a generic parametric family of probability distributions, the ***b*** parameter controls the shape such as for instance the symmetry of the distribution, while the scale parameter ***K***, accounts for the spreading of the distribution. Furthermore, a definite mode exists only for ***b ≥ 1*** and equals **(b-1) K**. Moreover, in the ***x***-domain the gamma-distribution can account for both symmetrical and asymmetrical distributions (enabling the modelling of a wider landscape of domains) and its Laplace transform in the ***λ***-domain is simply:

$\boldsymbol{f}_{\boldsymbol{0}}\left( \boldsymbol{\lambda} \right)\boldsymbol{=}\left( \boldsymbol{1+K\lambda} \right)^{\boldsymbol{-b}}$ (22)

so that rearranging Suppl. Eq.21 considering Supp. Eq. 18 and Suppl. Eq.15 as well as expressing the ***b*** and ***K***parameters as a function of $\bar{\boldsymbol{x}}$and $\boldsymbol{\sigma}^{\boldsymbol{2}}$ of ***Ψ(x)*** respectively, a suitable function to be fitted to the experimental dose-response curves is:

$\frac{\boldsymbol{\Delta I}}{\boldsymbol{I}_{\boldsymbol{0}}}\boldsymbol{=}\boldsymbol{A}_{\boldsymbol{sat}}\left( {\boldsymbol{1-}\left( \boldsymbol{1+}\frac{\boldsymbol{\sigma}^{\boldsymbol{2}}}{\bar{\boldsymbol{x}}}\boldsymbol{\cdot}\frac{\boldsymbol{c\cdot V\cdot}\boldsymbol{N}_{\boldsymbol{A}}}{\boldsymbol{n}} \right)}^{\boldsymbol{-}\left( \frac{\bar{\boldsymbol{x}}}{\boldsymbol{\sigma}} \right)^{\boldsymbol{2}}} \right)$ (23).

The fitting of the dose curves in Fig. 3a and Fig. 4a in the main text with Suppl. Eq.23 are shown by solid-red-lines. Indeed, the analytical model here produced, very accurately describes the experimental data. The best fit for the average $\bar{\boldsymbol{x}}$and the variance $\boldsymbol{\sigma}^{\boldsymbol{2}}$of the distribution of the number of binding sites per domain are the followings: $\bar{x}$ = 4 · 10^11^ and σ = 4 ·10^11^ for the gate functionalized with the whole SAM measured in PBS (dose-response-curve in Fig.3a in the main text); $\bar{x}=1\cdot{10}^{9}$ and σ = 4$\cdot$10^11^ results for the gate comprising only the physisorbed bio-SAM (dose-response-curve in Fig.4a in the main text). These valuesenabled to reveal the ***Ψ(x)*** distribution for a given SAM from its dose-curve and the actual ***Ψ(x)*** distributions for the dose-response-curves of Fig. 3a and Fig. 4a in the main text are given in Supplementary Fig. 11.


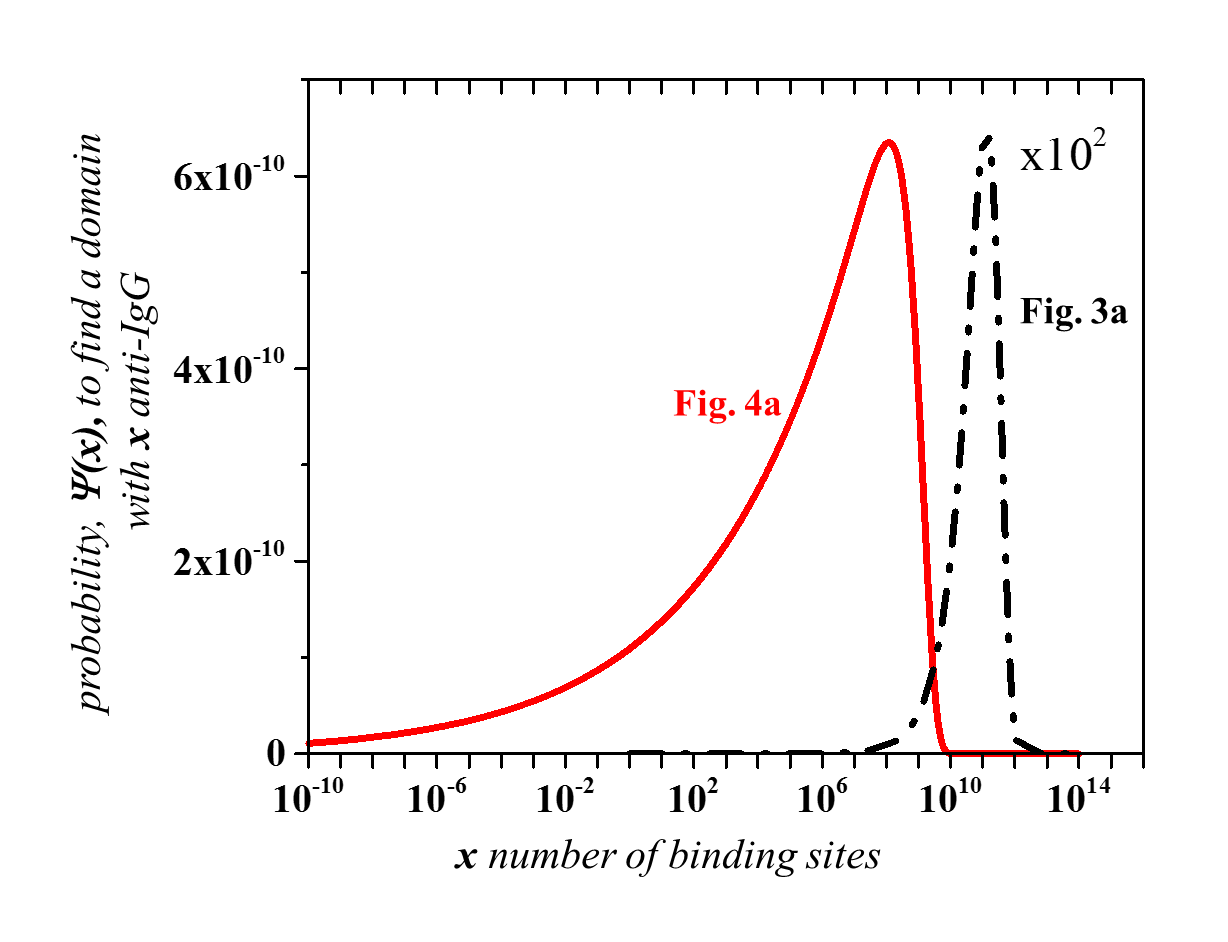


**Supplementary Figure 11:***Theprobability to find* ***x*** *binding sites per domain,* ***Ψ(x),*** *vs. the number of binding sites per domain,* ***x****.* ***Ψ(x****) values have been evaluated according to the gamma-distribution (Suppl. Eq.23) using the best fit parameters for a given dose curve. The broken blacklineis the* ***Ψ(x)*** *for the dose-response-curve in Fig. 3a, while the solid red line is relevant to the dose-curves of Fig.4a.*

The comparison between the extracted dispersion functions ***Ψ(x)*** for the two dose curves modeled is instructive as it evidences the predictive power of the SiMoT model. Among the system studied the more defect-free SAM comprises both the chemical and the biological component. Hence, for the SAM of the sensing in Fig. 3a there is a probability larger than 99% to find domains including as much as 10^11^ anti-IgG. This clearly evidences how the SAM is acting as amplification system of the single binding event as very large domains in which  changes are created. It is also apparent that, when the chem-SAM is not included the domains as shown for the modelling of the data in Fig. 4a in the main text, are two orders of magnitude smaller domains can be generated (96% probability) due to a less electrostatically connected system.

The compact nature of the bio-SAM is also important to achieve the single molecule detection and in this respect critical is the role of the anti-IgG surface blocking with the BSA. However, when more regularly shaped capturing proteins are used instead of the anti-IgG, the effect of the bio-blocking process is not critical anymore. This aspect will be addressed in another publication.

**Supplementary Note 8: The effect of the gating solution ionic-strength on the SiMoT response**

IgG sensing measurement is here performed on the SAM gate using a SiMoT gated with an electrolyte at different ionic strengths (i_S_). An *I_0_* base-line for each ionic strength of the electrolyte was measured by positioning the SAM-gate electrode in contact with the HPLC-grade water and then with NaCl solutions (0.01 mM, 0.05 mM, 0.1 mM, 10 mM, 100 mM and 200 mM) used as electrolyte gating-medium. The SAM gate was then incubated for 10 min in 100 μl of the PBS standard-solution of the ligands (IgG) with nominal concentrations of 6.67 10^8^ zM. After incubation in the PBS standard-solution of IgG the SAM was washed thoroughly with PBS first and then with water to remove the unreacted ligands away and further I-V transfer curves were measured in water. Sequentially,the same SAM was measured in the remaining six NaCl solutions at different ionic strength. The ΔI/I_0_ fractional changes has been evaluated for each ionic strength of the electrolyte medium. The data are plotted as a function of the Debye length (λ_D_) calculated λ_D_ = 0.303 (i_S_)^-0.5^.^[[35]](#endnote-34)^


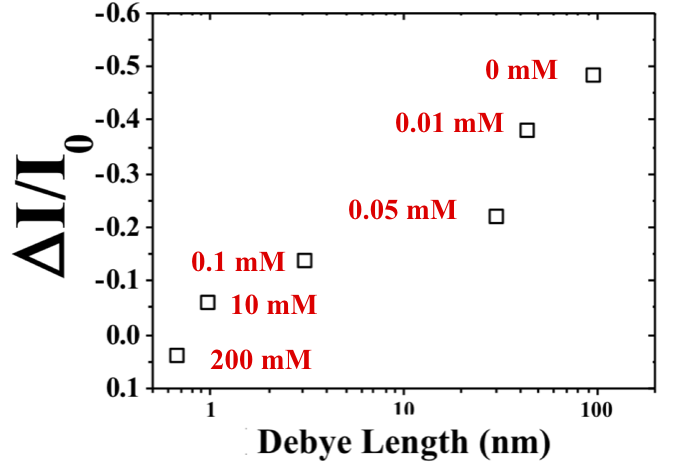


**Supplementary Figure 12*:*** *Fractional changes of the current (ΔI/I_0_) as a function of the Debye Length in an NaCl solution.*

Apparently, the fractional change of I_D_ drops by approximately a factor two for λ_D_< 30 nm and vanishes below 1 nm. Therefore, for λ_D_< 3 nm the detection is impaired. This experiment very well reproduces the expected trend^[[36]](#endnote-35),^^[[37]](#endnote-36)^ and provides the rationale to operate the SiMoT in pure water, where λ_D_≈ 100 nm namely is long enough to make sure that all SAM which is only few nm high, is not shielded and all the electrostatic changes can be accurately measured. This experiment further corroborates the already proven ion-permeable nature of the bio-SAM.

**Supplementary Note n 9. Quantification of endogenous human IgG in whole saliva sample**

The human saliva sample from a healthy female volunteer was diluted with PBS to obtain a stock solution of saliva in a ratio of 1:100. To quantify the endogenous content of IgG minimize matrix effects, the standard addition method was used.To this end, stock solutions containing the same amount of diluted saliva were spiked with known amounts of human IgG to yield spiked saliva samples with standards concentration in the range 0.1 – 1 nM. The total amount of IgG in human saliva (endogenous + spiked) was measured by means of SPR measurements performed by loading aliquots (100 μL) of IgG-spiked human saliva to a gold-coated glass SPR slide previously functionalized with the anti-IgG containing SAM. The SAM antibody-coated SPR slides were incubated with the analyte solution at 20°C until a plateau of the SPR signal was reached and it was thoroughly rinsed afterwards with aliquots of buffer to remove any loosely bound proteins. The sensograms were obtained by recording the reflection angle as a function of time at 670 nm. All measurements were taken in triplicate using different SPR immune sensors. Suppl. Fig. 13 shows the calibration curve as the change in the angle versus the concentration of added IgG in human saliva. The range of concentration shown is limited to the range where the response is linear with the concentration of the analyte. The calibration graphs were used for direct determination of IgG in human saliva yielding a concentration of endogenous IgG of 40 ± 6 nM. The standard deviation for the extrapolated value was calculated according to reference.^[[38]](#endnote-37)^


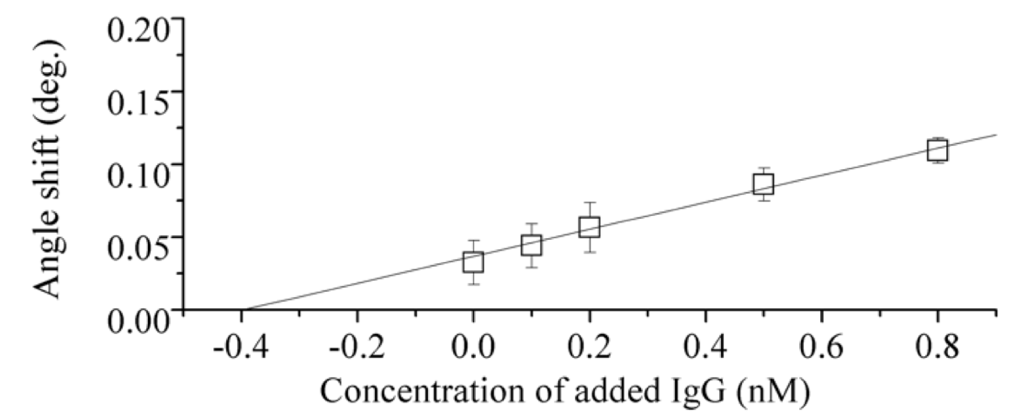


**Supplementary Figure 13**: *Calibration curves of the IgG at different concentrations in diluted human saliva, binding to 100 μg/ml anti-IgG immobilized onto Au slide*

**Supplementary Note 10. Negative control experiment in BSA**

The data in Fig. 3a in the main text are completed with the data shown in the case is being analysed by the editors and a response will be provided as soon as possible. Supplementary Fig.14. Here a gate functionalized with the SAM is incubated for ten minutes for nine subsequent times in bare PBS and the transfer characteristic is measured after every incubation. The ΔI/I_0_ is computed for each incubation and the data are shown in Suppl. Fig. 14. The average ΔI/I_0_ is 0.01±0.01 and this can be taken as the reproducibility error of the SiMoT response in a negative control experiment.


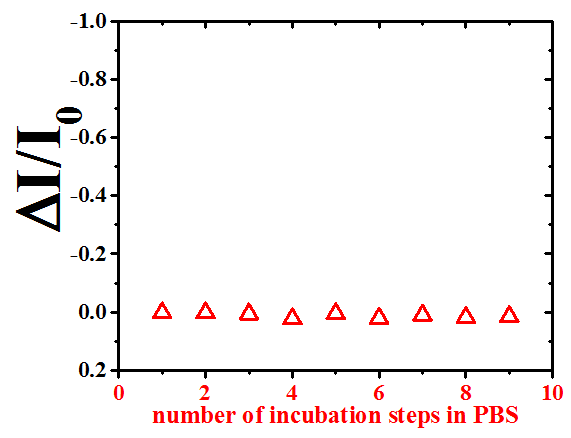


**Supplementary Figure 14:** *I_D_ current relative variations after nine subsequent incubations (for ten minutes each time) of the same SAM gate in a bare PBS solution.*

**Supplementary Note 11. Molecular modelling of the chem-SAM**

***Density functional theory calculations***

****** To reconstruct the gold surface, a bulk-gold super cell was optimized by means of periodic density functional theory (DFT), obtaining a cell parameter a = 4.152 Å close to the experimental one (a_exp_= 4.078 Å).^[[39]](#endnote-38)^ The Au(111) surface was cut from this super cell and optimized keeping the gold atoms belonging to the lowest layer fixed so as to simulate bulk constraints. EDC/sulfo-NHS chemical activated and blocked with ethanolamine 3-MPA were let to absorb onto the reconstructed gold surface (Fig.15S) in the (√3×√3)R30° configuration, already proven to be the most stable for these systems.^[[40]](#endnote-39),^^[[41]](#endnote-40),^^[[42]](#endnote-41)^

**Supplementary *Figure 15:*** *2D sketch of an activated and blocked 3-MPA SAM attached on a gold 111 surface. The angle* ** ***is detailed in red while the H-bonds, occurring between adjacent chains, are itemized by dashed lines.*

The resulting DFT optimized structure is shown in Suppl. Fig.16. The added functional groups and the reorientation occurred after the EDC/sulfo-NHS chemical activation and the blocking in ethanolamine of the 18 3-MPAs enabled the formation of an H-bond network evidenced in Supp. Fig.16.


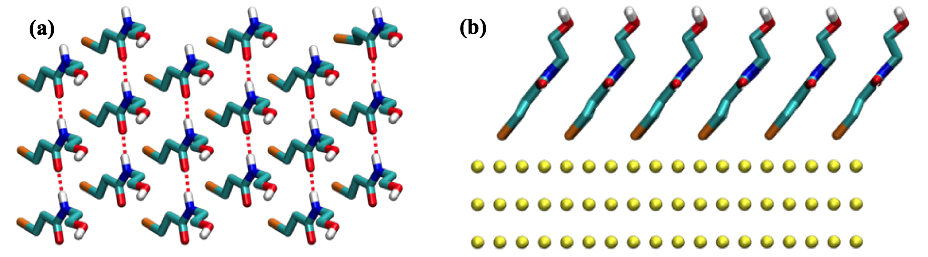


**Supplementary *Figure 16:*** *DFT optimized structure of the activated and blocked 3-MPA: top x-y (a) and side (b) views. The H-bondnetwork is evidenced by red-dashed lines. For the sake of clarity non-polar hydrogen (top and side views) as well as gold atoms (top view) are not shown.*

***Molecular dynamics simulations***

Molecular dynamics (MD) simulations have been performed on a larger cell. In particular, the system was built as follows: - the DFT optimized configuration was replicated generating a cell comprising 6480 activated and blocked 3-MPA chains; - 589 3-MPA chains were replaced by EDC/sulfo-NHS chemical activated and blocked with ethanolamine 11-MUA to reproduce the experimental conditions (molar ratio 3-MPA /11-MUA equal to 10:1). The final system comprising 143.856 atom is shown in Supplementary Fig.17.


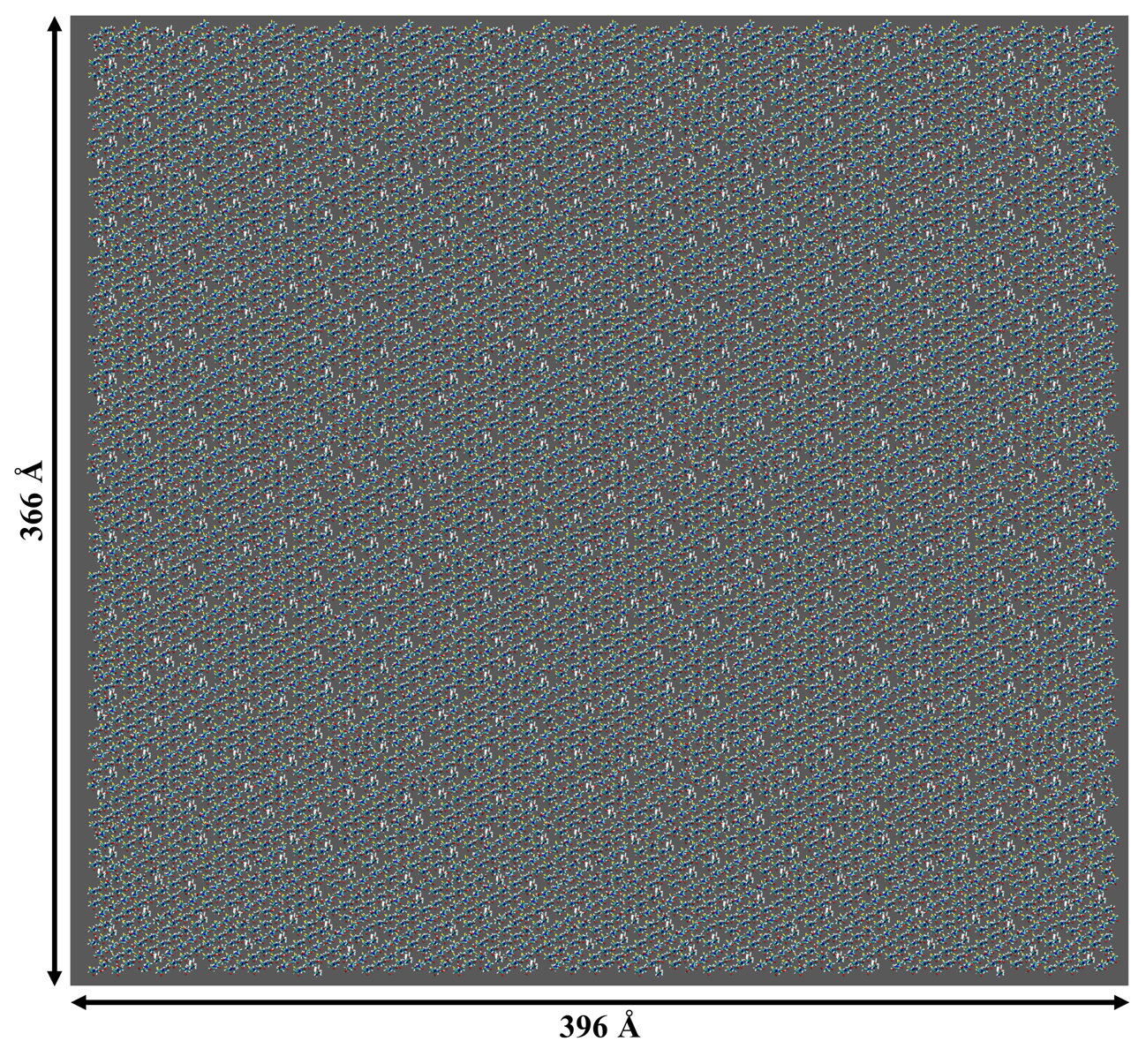


**Supplementary *Figure 17:*** *Top view of the simulated system; the x-y plane is 396 Å x 366 Å wide and it contains 5892 activated and blocked 3-MPA and 589 activated and blocked 11-MUA chains.*

All simulations were performed at T = 25 °C and under the following different conditions: - **i)** in implicit solvent (water) setting the dielectric constant ε = 80; - **ii)** in the same condition as **i)** but in the presence of a defect generated by restraining the coordinates of a region (force constant k = 1 kcal mol^-1^ Å^-2^) with a radius of 12 Å in a disordered conformation (missing of H-bonds); - **iii)** as in **i)** but in the presence of an uniformly applied electric field (E = - 0.1 V/nm to simulate the field generated in the SAM by the gate potential) oriented in the z-axis direction perpendicular to the SAM surface and setting the dielectric constant ε = 6 to account for the charge double layer; **iv)** as in **ii)** but in the presence of elicited electric field as well as of the disordered conformation and also with ε = 6.

As a first step, the obtained 100-ns long trajectories were analysed by computing the nematic order parameter *P_2_* and the dynamic order parameter *S,* commonly used in MD simulations to assess the orientational order of complex chemical systems.^[[43]](#endnote-42),^^[[44]](#endnote-43),^^[[45]](#endnote-44)^ The former is defined in Supplementary Eq. 24, as the largest eigenvalue of the Q_ab_ tensor:

$Q_{ab}=\left\langle\left. \frac{1}{N}\sum_{i=1}^{N} \left( \frac{3}{2}\hat{u}_{ia}\hat{u}_{ib}-\frac{1}{2}\delta_{ab} \right) \right\rangle\right.$ (24)

where the angle brackets indicate the average over the analysed trajectory, $\delta_{ab}$ is the Kronecker delta and *N* corresponds to the number of vectors $\hat{u}_{i}$. For each SAM chain *i,*$\hat{u}_{ia}$ corresponds to the components a = x,y,z of the unit vector starting from the amidic hydrogen atom and pointing towards the amidic oxygen atom, thus defining the orientation of the single chain. P_2_ ranges from 0 to 1. In particular, P_2_ = 1 indicates a totally ordered system, while a value of P_2_ = 0 corresponds to a total disorder system. S describes the stability in time of the order indicated by P_2_ and is computed as follows (Suppl. Eq. 25):

$S=\left\langle\left. \frac{1}{N}\sum_{i=1}^{N} \left( \frac{3}{2}{(\hat{U}}_{i}\cdot\hat{u}_{i})^{2}-\frac{1}{2} \right) \right\rangle\right.$ (25)

where $\hat{U}_{i}=\left\langle\hat{u}_{i} \right\rangle$. S ranges from - 0.5 to 1. In particular, S = 1 implies a constant orientation, while a smaller S value indicates that the orientation of the chains is changing over time. The obtained order parameters, shown in Supplementary Tab. 5, shed light on the effect of the applied electric field on the simulated system. Indeed, P_2_ computed for simulations **iii** and **iv** are significantly higher with respect to those resulting from simulations **i** and **ii**, thus indicating that the presence of an electric field is necessary for generating an ordered system. Such a trend is confirmed if S is considered, being the obtained values negative only in the absence of an electric field (simulations **i** and **ii**).

**Supplementary *Table 5:*** *Angle* *******, Nematic (P_2_) and dynamic (S) order parameters computed for all the performed MD simulations (i, ii, iii and iv).*

|  | ***i) before binding***  ***E = 0*** | ***ii) after binding***  ***E = 0*** | ***iii)before binding***  ***E = 0.1 V/nm*** | ***iv) after binding***  ***E = 0.1 V/nm*** |
| --- | --- | --- | --- | --- |
| **** | 26.15 ± 0.16 | 26.01 ± 0.17 | 33.48 ± 0.12 | 28.07 ± 0.14 |
| **P_2_** | 0.30 ± 0.01 | 0.21 ± 0.01 | 0.58 ± 0.01 | 0.69 ± 0.01 |
| **S** | -0.13 ± 0.01 | -0.34 ± 0.01 | 0.76 ± 0.01 | 0.47 ± 0.01 |

From a geometrical point of view such a finding can be rationalized by comparing the values averaged along the MD trajectories of the angle ****, defined by the vector originating from the sulfur atom and pointing towards the oxydril oxygen atom of activated and blocked 3-MPA chain and the z-axis normal to the gate surface plane and aligned with the gating field (Suppl. Fig. 15). In the absence of an applied electric field, the performed simulations return ** ** values equal to 26.15 ± 0.16 degrees (simulation **i**) and 26.01 ± 0.17 degrees (simulation **ii**). Significantly higher values result from simulations **iii** (33.48 ± 0.12 degrees) and **iv** (28.07 ± 0.14 degree).

The obtained 100-ns long trajectories resulting from simulations **iii** and **iv** were also analysed focusing the attention on the direction and relative occurrence (%) of the detected H-bonds in the system in order to get insights into the electric field driven order (Suppl. Fig. 18 and Suppl. Fig. 19 or Fig. 4c and Fig. 4d in the main text). It is worth noting that the H-bonds are visualized with arrows originating from the amide hydrogen atom involved in the H-bond interaction and pointing towards the H-bond acceptor atom (amide oxygen). The colour-code indicates the percentage of frames in which the hydrogen bond is formed, using as thresholds a distance from the acceptor atom (AA) to the donor atom (AD) equal to 3 Å and an angle AD - H - AA equal to 150°. Importantly, a visual comparison of Suppl. Fig. 18 and Suppl. Fig. 19 reveals that the imposed disordered conformation in a small region having a radius of 12 Å generates a new H-bond pattern in the whole system.

**
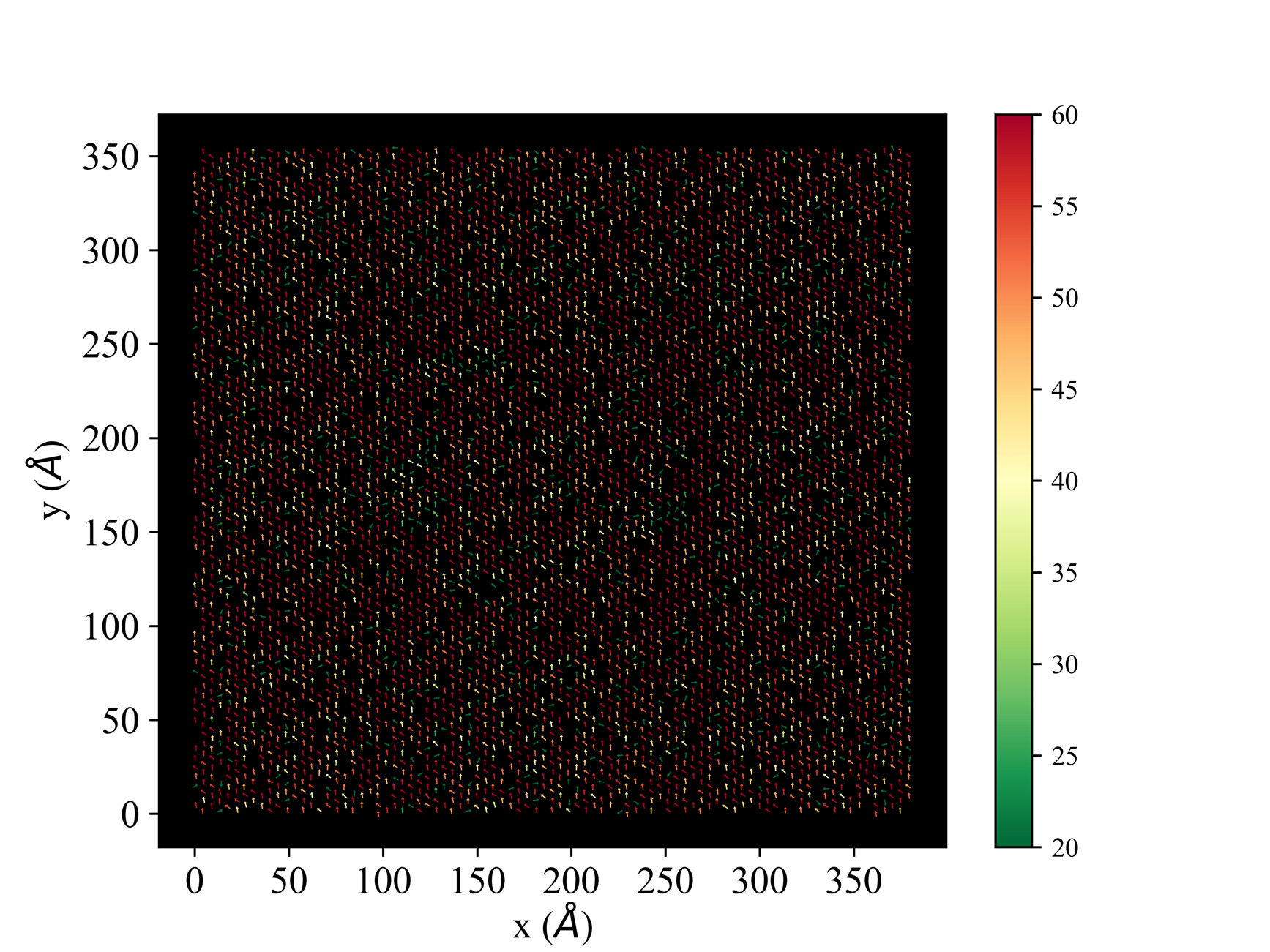
**

**Supplementary *Figure 18****: Direction of the H-bonds detected during the 100 ns-long MD simulation of the system at RT and under the gating-field (simulation* ***iii****). The color-codes indicate the percentage of frames in which the H-bond is established.*

*
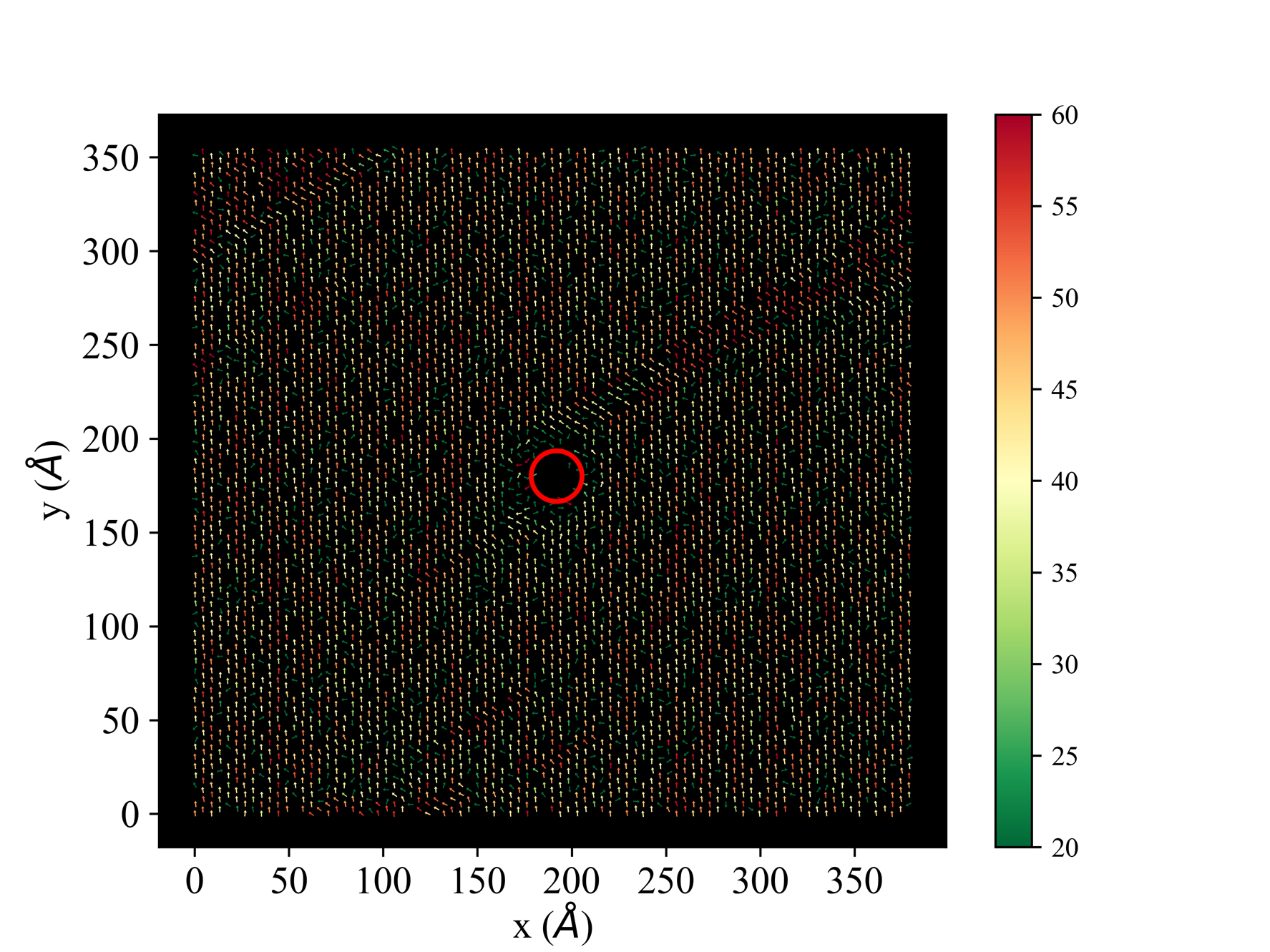
*

**Supplementary *Figure 19****: Direction of the H-bonds detected during the 100 ns-long MD simulation of the system at RT, under the gating-field and after a binding event simulated by means of a defect obtained by imposing a disordered conformation (missing of an H-bond network) to a region with a radius of 12 Å (simulation* ***iv****) defined by the red circle. The color-codes indicate the percentage of frames in which the H-Bond is established.*

**Supplementary References**

1. *^§^*Present address: IstitutoTumori IRCCS Giovanni Paolo II, Viale O. Flacco 65, 70124 - Bari (I) [↑](#footnote-ref-1)
2. Cheng, S. et al. Field effect transistor biosensor using antigen binding fragment to detect tumor marker in human serum. *Materials***7**, 2490 (2014). [↑](#endnote-ref-1)
3. Zhong C., Zhong-Liang X., Ling Z., Dong-Mei L., Masao K., Maki S. Molecule counting with alkanethiol and DNA immobilized on gold microplates for extended gate FET. *Materials Science and Engineering C***33**, 1481 (2013). [↑](#endnote-ref-2)
4. Hideshima S., Sato R., Inoue S., Kuroiwa S., Osakaa T. Detection of tumor marker in blood serum using antibody-modified field effecttransistor with optimized BSA blocking. *Sensors and Actuators B* **161**, 146 (2012). [↑](#endnote-ref-3)
5. Worm J. Winspall 3.02.http://www.mpip-mainz.mpg.de/groups/knoll/softwar., (2012). [↑](#endnote-ref-4)
6. Silverton E. W., Navia M. A., Davies D. R. Three-dimensional structure of an intact human immunoglobulin. *Proc. Natl. Acad. Sci. U.S.A.***74**, 5140 (1977). [↑](#endnote-ref-5)
7. Xu H., Lu J.R. and Williams D.E. Effect of SurfacePackingDensity of InterfaciallyAdsorbedMonoclonalAntibody on the Binding of HormonalAntigen Human ChorionicGonadotrophin. J. Phys. Chem. B **110**, 1907 (2006). [↑](#endnote-ref-6)
8. Vörös J. The densityandrefractiveindexofadsorbingproteinlayers. *Biophys. J.***87**, 553 (2004). [↑](#endnote-ref-7)
9. Yu C., Irudayaraj J. Quantitative Evaluation ofSensitivityandSelectivityof Multiplex NanoSPR Biosensor Assays. *Biophys. J.***93**, 3684 (2007). [↑](#endnote-ref-8)
10. Casalini, S., Dumitru, A.C., Leonardi, F., Bortolotti, C.A., Herruzo,E.T., Campana, A., de Oliveira, R.F., Cramer, T., Garcia, R. &Biscarini,F. Multiscale Sensing of Antibody–Antigen Interactions by Organic Transistors and Single-Molecule Force Spectroscopy. *ACS Nano***9**, 5051-5062 (2015). [↑](#endnote-ref-9)
11. Trilling, A. K., Beekwilder, J. &Zuilhof, H. Antibody Orientation on Biosensor Surfaces: A Minireview. *Analyst***138**,1619–1627 (2013). [↑](#endnote-ref-10)
12. Le Brun, A. P., Holt, S. A., Shah, D. S. H., Majkrzak, C. F. &Lakey, J. H. The Structural Orientation of Antibody Layers Bound to Engineered Biosensor Surfaces. *Biomat.***32**, 3303 (2011). [↑](#endnote-ref-11)
13. Wentworth, P., Wentworth, A.D., Zhu, X., Wilson, I.A., Kim, D., Janda, K., Eschenmoser, A. & Lerner, R.A. Evidence for the Production of Trioxygen Species During Antibody-Catalyzed Chemical Modification of Antigens. *Proceedings of the National Academy of Science of USA***100**, 1490–1493 (2003). [↑](#endnote-ref-12)
14. Jakešová, M., Apaydin, D.H., Sytnyk , M., Oppelt, K., Heiss,W., Sariciftci, N.S. &Głowacki, E.D. Hydrogen-BondedOrganicSemiconductorsasStablePhotoelectrocatalysts for EfficientHydrogenPeroxidePhotosynthesis.*Adv. Funct. Mater.***26**, 5248–5254 (2016). [↑](#endnote-ref-13)
15. Lasia, A., *Electrochemical Impedance Spectroscopy and its Applications.*Modern Aspects of Electrochemistry, **32**, Springer, Boston, MA (2002). [↑](#endnote-ref-14)
16. Noriega, R. *et al.* A general relationshipbetweendisorder, aggregation and chargetransport inconjugatedpolymers. *Nat. Mater.* **12,** 1038–1044 (2013). [↑](#endnote-ref-15)
17. Street, R. A., Northrup, J. E. &Salleo, A. Transport in polycrystallinepolymerthin-film transistors. *Phys. Rev. B* **71,** 165202 (2005). [↑](#endnote-ref-16)
18. Salleo, A. *et al.* Intrinsicholemobility and trapping in a regioregular poly(thiophene). *Phys. Rev. B* **70,** 115311 (2004). [↑](#endnote-ref-17)
19. Salleo, A., Kline, R. J., DeLongchamp, D. M. &Chabinyc, M. L. Microstructuralcharacterization and chargetransport in thinfilms of conjugatedpolymers. *Adv. Mater.* **22,** 3812–3838 (2010). [↑](#endnote-ref-18)
20. Chang, J. F., Sirringhaus, H., Giles, M., Heeney, M. &McCulloch, I. Relative importance of polaronactivation and disorder on chargetransport in high-mobilityconjugatedpolymerfield-effecttransistors. *Phys. Rev. B* **76,** 205204 (2007). [↑](#endnote-ref-19)
21. Wang, C. *et al.* Microstructuralorigin of high mobility in high-performance poly(thieno-thiophene) thin-film transistors. *Adv. Mater.* **22,** 697–701 (2010). [↑](#endnote-ref-20)
22. MacKenzie, R. C. I., Shuttle, C. G., Chabinyc, M. L. & Nelson, J. Extractingmicroscopicdeviceparameters from transientphotocurrentmeasurements of P3HT:PCBM solar cells. *Adv. Energy Mater.* **2,** 662–669 (2012). [↑](#endnote-ref-21)
23. Torricelli, F. *et al.* Unified drain-current model of complementary p- and n-type OTFTs. *Org. Electron.* **22,** 5–11 (2015). [↑](#endnote-ref-22)
24. Hong, K., Kim, S. H., Mahajan, A. &Frisbie, C. D. Aerosol jet printed p- and n-typeelectrolyte-gatedtransistors with a variety of electrodematerials: Exploringpracticalroutes to printedelectronics. *ACS Appl. Mater. Interfaces* **6,** 18704–18711 (2014). [↑](#endnote-ref-23)
25. Northrup, J. E. Atomic and electronicstructure of polymerorganicsemiconductors: P3HT, PQT, and PBTTT. *Phys. Rev. B* **76,** 245202 (2007). [↑](#endnote-ref-24)
26. Kergoat, L. *et al.* A water-gate organicfield-effect transistor. *Adv. Mater.* **22,** 2565–2569 (2010). [↑](#endnote-ref-25)
27. Melzer, K. *et al.* Characterization and simulation of electrolyte-gatedorganicfield-effecttransistors. *Faraday Discuss.* **174,** 399–411 (2014). [↑](#endnote-ref-26)
28. Rivnay, J. *et al.* Structuralorigin of gap states in semicrystallinepolymers and the implications for chargetransport. *Phys. Rev. B* **83,** 121306(R) (2011). [↑](#endnote-ref-27)
29. Štêpánek, P., Data Analysis in Dynamic Light Scattering, in Dynamic Light Scattering: The Method and Some Applications. Chapter 4, author Brown, W. Ed., Oxford Science Publications, Clarendon Press, Oxford, UK (1993**).** [↑](#endnote-ref-28)
30. Austin, R. H., K. W. Beeson, L. Eisenstein, H. Frauenfelder, and I. C. Gunsalus. Dynamics of ligand binding to myoglobin. *Biochemistry* **14**, 5355–5373 (1975). [↑](#endnote-ref-29)
31. Kleinfeld, D., M. Y. Okamura, and G. Feher. Electron transfer kinetics in photosynthetic reaction centers cooled to cryogenic temperatures in the charge-separated state: evidence for light-induced structural changes. *Biochemistry***23**, 5780–5786 (1984). [↑](#endnote-ref-30)
32. G. Palazzo, A. Mallardi, A. Hochkoeppler, L. Cordone, G. Venturoli. Electron Transfer Kinetics in Photosynthetic Reaction Centres Embedded in Trehalose Glasses: Trapping of Conformational Substates at Room Temperature. *Biophys. J.***82**, 558-568 **(**2002**).** [↑](#endnote-ref-31)
33. F. Francia, M. Dezi, A. Mallardi, G. Palazzo, L. Cordone, G. Venturoli. Protein-Matrix Coupling/Uncoupling in “Dry” Systems of Photosynthetic Reaction Centre Embedded in Trehalose/Sucrose: The Origin of Trehalose Peculiarity. *J. Am. Chem. Soc.***130**, 10240-10246 **(**2008**).** [↑](#endnote-ref-32)
34. Weisstein, E.W. in*CRC Concise Enciclopedia of Mathematics*, Chapman & Hall/CRC, New York, (1999), p. 694. [↑](#endnote-ref-33)
35. Israelachvili, J., *Intermolecular and Surface Forces*. **14**, Academic Press, London, UK (1992). [↑](#endnote-ref-34)
36. Kim A. *et al.* Direct label-free electrical immunodetection in human serum using a flowthrough-

    apparatus approach with integrated field-effect transistors. *Biosens. Bioelectron***25,** 1767 (2010). [↑](#endnote-ref-35)
37. Stern, E. *et al.* Label-free biomarker detection from whole blood.*Nat. Nanotechnol.***5**, 138 (2010). [↑](#endnote-ref-36)
38. Miller,J. N. &Miller,J. C. Statistics and Chemometrics for Analytical Chemistry. Sixth Edition 2010,Pearson Education Limited 2000, 2010 Harlow- England, p.129. [↑](#endnote-ref-37)
39. Wyckoff, R. W. G. Crystal Structures. (Wiley, 1963) [↑](#endnote-ref-38)
40. Mrksich, M. &Whitesides, G. M. Using Self-Assembled Monolayers That Present Oligo(ethylene

    glycol) Groups To Control the Interactions of Proteins with Surfaces. in Poly(ethylene glycol) **680**,

    361–373 (American Chemical Society, 1997) [↑](#endnote-ref-39)
41. Yang, Y.-C., Chang, T.-Y. & Lee, Y.-L. Adsorption Behavior of 11-Mercapto-1-undecanol on

    Au(111) Electrode in an Electrochemical System. J. Phys. Chem. C **111**, 4014–4020 (2007). [↑](#endnote-ref-40)
42. Liu, Y.-F. & Lee, Y.-L. Adsorption Characteristics of OH-Terminated Alkanethiol and Arenethiol

    On Au(111) Surfaces. Nanoscale**4**, 2093–2100 (2012). [↑](#endnote-ref-41)
43. Komolkin, A.V., Laaksonen, A. and Maliniak, A. Molecular dynamics simulation of a nematic liquid crystal. *J. Chem. Phys*. **101**, 4103-4116 (1994). [↑](#endnote-ref-42)
44. Alberga, D., Mangiatordi G.F., Torsi L., Lattanzi, G. Effects of Annealing and Residual Solvents on Amorphous P3HT and PBTTT Films.*J. Phys. Chem. C***118**, 8641-8655 (2014). [↑](#endnote-ref-43)
45. Alberga D., Perrier A., Ciofini I., Mangiatordi G.F., Lattanzi G., Adamo C. Morphological and charge transport properties of amorphous and crystalline P3HT and PBTTT: insights from theory. *Phys. Chem. Chem. Phys.***17**, 18742-18750 (2015). [↑](#endnote-ref-44)
